# Can valence and origin of emotional words influence the assessments of ambiguous stimuli in terms of warmth or competence?

**DOI:** 10.7717/peerj.10488

**Published:** 2021-01-26

**Authors:** Kamil K. Imbir, Maciej Pastwa

**Affiliations:** Faculty of Psychology, University of Warsaw, Warsaw, Poland

**Keywords:** Emotional words, Valence, Origin of affect, Ambiguous task processing, Social cognition

## Abstract

People tend to think that emotions influence the way they think in a spectacular way. We wanted to determine whether it is possible to prime the assessments of ambiguous stimuli by presenting emotion-laden words. We did not expect the differences in assessments that depend only on the emotional factors to be particularly large. Participants were presented with words differing in valence and origin of an affective state, but aligned for arousal, concreteness, length and frequency of use. Their first task was to remember a word. While keeping the word in mind, their second task was to guess by intuition whether the symbol was related to certain traits. Participants assessed objects represented by coding symbols on the scales of warmth or competence. We expected positive valence and automatic origin to promote higher ratings in terms of warmth and reflective origin to promote higher ratings in terms of competence. Positive valence appeared to boost assessments in terms of both warmth and competence, while the origin effect was found to be dissociative: automatic origin promoted intensity of warmth assessments and reflective origin intensity of competence assessments. The study showed an existing relation between emotional and social aspects of the mind, and therefore supports the conclusion that both domains may result from dual processes of a more general character.

## Introduction

Information from the environment can be implicitly processed (Greenwald, McGhee & Schwartz, 1998) and influence the way we think and judge (Ferguson, Bargh & Nayak, 2005; Payne et al., 2010; Payne & Lundberg, 2014). Such an influence is also ascribed to affective properties of surrounding stimuli and is defined as affective priming (Murphy & Zajonc, 1993; Wentura, Rohr & Degner, 2017) or affect misattribution (Blaison et al., 2012; Payne et al., 2005; 2010). The fundamental question concerns whether aspects of priming stimuli other than valence may influence assessment of the neutral stimuli. In this article, we report on the investigation concerning the valence and origin of an affective state, the two distinct dimensions ascribing emotional reactions to verbal stimuli (Imbir, 2016a, 2016c; Jarymowicz & Imbir, 2015), and assess neutral stimuli from the perspective of social cognition.

### Fundamental dimensions of social cognition and dual-mind perspective

Social cognition is the term proposed for labelling the cognitive processes that are focused on a social environment (Fiske, Cuddy & Glick, 2007). The themes of cognition may range from simply other people, understood as more or less important figures in ones’ life, to preconceptions about internal/mental characteristics of people which can lead to conclusions about their motivations. Relations between people are a very important topic of social cognition, as it is in relations that people express their internal traits and motivations. It has been found that there are two distinct dimensions that account for the span of social cognition: warmth and competence (Abele & Wojciszke, 2007; Fiske, Cuddy & Glick, 2007). Warmth describes people’s intentions towards us. ‘Warm’ people are trustworthy, helpful, sincere, honest, sociable and so on. The opposite pattern is associated with ‘cold’ people. Competence describes people’s abilities to turn intentions into actions. The competent person is clever, creative, efficient, foresighted, ingenious, intelligent and knowledgeable (Fiske, Cuddy & Glick, 2007; Ponsi et al., 2016).

Both dimensions refer to distinct characteristics which explain the reasons for the interpretation and understanding of the vast majority of other people’s observed behaviours, which has been frequently proven (Abele & Wojciszke, 2007; 2014). Warmth and competence have turned out to be the traits that primarily shape the interpretation of briefly presented, unknown faces (Willis & Todorov, 2006). The assessments of socially perceived ability, which could be understood as competence, have been proven to interact with the approach and avoidance tendencies (Peeters, 2002), while perceived warmth stimulates inferences about the motives of other people (Reeder et al., 2002). Warmth has been found to be more important than competence and is therefore prioritised in processing (Fiske, Cuddy & Glick, 2007; Imbir, 2017a, 2017c; Willis & Todorov, 2006). For example in lexical decisions, it was found that warmth-related words were distinguished from pseudo words faster than were competence-related words (Ybarra, Chan & Park, 2001). From an evolutionary point of view, thinking more in terms of warmth than competence is relevant because it is more important to get to know other people’s intentions (bad or good) than to have the ability to make intentions true (high or low). It also seems easier to interpret warmth than competence. The definition of warmth is based on characteristics that can be easily experienced in relation to another person (i.e. helpfulness, honesty, sociability, understanding, etc.), while the characteristics of competence are more abstract and based on the cognitive abilities of others (like cleverness, creativity, efficiency, foresight, ingenuity, intelligence, knowledgeability, etc.). This gives us a hint that warmth and competence in social cognition may be related to the dual-mind perspective in psychology (Epstein, 2003; Gawronski & Creighton, 2013; Kahneman, 2011).

The duality-of-mind perspective is a group of theories that explain cognitive processes in terms of two complementary modes operating with the use of distinct rules (Epstein, 2003; Strack & Deutsch, 2004): it is based on experiential vs rational processing. Experiential processing is based on associative mechanisms and thus means slow learning even though it appears effortless and fast. When we are forced to assess something immediately, the best solution is to learn repetitive patterns of stimulus, allowing us to choose the best responses (Strack & Deutsch, 2004). The response selection in associative mode is based on a complex pattern of perceived traits, their relations and predictive values and can, therefore, be a complex process (Strack & Deutsch, 2014), but it is automatic and therefore effortless in giving an approximation of the correct response (Epstein, 2003; Kahneman, 2011; Slovic et al., 2002). The warmth dimension analysis suggests that this dimension may be more experiential, especially because it is prioritised in terms of time and more visible on the facial-expression level (Willis & Todorov, 2006; Ybarra, Chan & Park, 2001). Assessing warmth brings information about the possible threat coming from other person, which is crucial for initiating an interaction. Rational processing is based on algorithmic execution of all cognitive operations needed to get the correct answer to a problem. Such operations are language based, propositional and located in a limited resources space (like an operating memory). The competence dimension is also less visible to observers, because it is based on the hidden mechanism of thinking, planning, efficiency in realising aims, etc. This analysis suggests that the competence dimension should be more associated with the rational mind. The assessment of competence answers a question whether a person is capable of managing a certain goal, no matter if this goal is desired or not by the person assessing.

In the duality-of-mind perspective, there is also a claim that affective processes may be based on processes that are characteristic of both experiential and rational minds (Imbir, 2016c; Jarymowicz & Imbir, 2015). The so-called ‘automatic emotions’ have been postulated as being immediate and direct affective reactions to stimuli (e.g. objects in the environment or intrinsic, bodily processes) which are uncontrolled and spontaneous. They evoke stereotypical reactions to situations that are repeated in human life. Damasio (2010) proposed biological value as a mechanism for the elicitation of such emotions. Everything that is good for the biological survival of an organism is pleasurable for this individual, while all threats are unpleasurable. This creates a universal motivational mechanism for even relatively simple organisms (Damasio, 2010; Damasio & Damasio, 2016). We should not neglect such mechanisms in humans and, what is more, intuitively defined emotions are of this type (Kagan, 2007). Even in the dual-mind theories, emotions are in most cases defined as automatic reactions to biologically meaningful stimuli, like fear being a reaction to the angry face staring at us (Epstein, 2003; Kahneman, 2011). Other examples of automatic emotions could be physical distress or calmness as well as ecstasy or disgust provoked, respectively, by arousing or repulsive stimuli (Jarymowicz & Imbir, 2015).

Reflective originated emotions are more complex than cognitively-based affective experiences (Jarymowicz, 2012). They are based on an elaborated analysis of the situation’s meaning in the context of an individual’s norms and expectations towards reality. We may claim that a certain idea is good for us or the world (like a vegetarian diet), and such a verbalised claim will drive the way we think about what food is tasty and what type of food to choose for dinner. This will modify emotional reactions based on biological value, but cognition is not as fast as automatic reactions are, therefore reflective emotions are more deferred in time; they also raise more doubts in us, resulting in a less subjective certainty of the validity of our experiences. There is a space for hesitation and for different perspectives (e.g. in dieting: ethical, economic, medical, etc.). The standards of evaluation are representations of knowledge and therefore may work only in a rational mind due to the propositional mechanisms (Strack & Deutsch, 2004). Examples of such emotions could be shame or self-acceptance, both deriving from the social standards (Jarymowicz & Imbir, 2015).

To measure automatic vs reflective originated emotional reactions, the Self-Assessment Manikin (SAM) scale was introduced (Imbir, 2015) based on the heart vs mind philosophical dichotomy widely distributed in Western culture. Automatic emotions were presented as heart associated, while reflective emotions were symbolised by the mind and consideration. The scale appeared to be reliable in terms of repeatability of results and, therefore, allowed the collection of affective norms for words (Imbir, 2015, 2016a), short texts (Imbir, 2016b, 2017b) and even music excerpts (Imbir & Goląb, 2017). Such stimuli are ideal in affective studies because we can provide a checked-in-advance list of stimuli that differ in critical dimensions but align in other dimensions and investigate the cognitive consequences of such a presentation.

Emotions can also be described in a different way, not divided into categories and differentiated from cognitive experiences but rather as an inseparable part of almost every experience. Based on the theory of constructed emotion (Barrett, 2017a; 2017b), we can describe a certain situation of assessing warmth or competence as a holistic process in which emotion shapes the prediction, thus promoting a certain action (assessment). The theory of constructed emotion suggests that emotions should not be classified into certain categories, as the feelings may strongly differ between individuals, activating different neural structures. Nevertheless, the author of the theory explicitly outlines the existence of automatic processes related to basic biological needs of an organism and deriving from limbic structures, as well as more systematic processes related to social concepts in which the prefrontal cortex plays the most important role (Barrett, 2017b). Based on the theory of constructed emotion, in a situation in which automating concepts promote automatic processing, including the assessment of warmth, the automating concepts would be seen as not only thematically but also structurally related, while reflective concepts would promote systematic thinking in an analogical relation. From this point of view, we would not speak about cross-system interactions but rather more complex processes involving larger amounts of neural structures, as they involve more differing themes. The outcome would still be similar—the decisions taking place in just one system would be easier and more certain, while more complex ones involving both systems would seem more difficult and would not be as certain.

### Ambiguous stimuli processing: the early evidence of emotion–social cognition interactions

The way we understand the surrounding environment may be affected by several factors, including affective state (Ferguson, Bargh & Nayak, 2005). The affect infusion in a decision-making process is quite unconscious and delicate but significant (Forgas, 1995; Murphy & Zajonc, 1993). Affect may alter the way we perceive objects that are not clearly interpretable or known in advance. This is why stimuli, such as Chinese ideograms, hexagrams and octagrams, become useful in paradigms based on their assessment on scales not related strictly to their look but rather to the traits to which the symbols may relate. In an original study by Murphy & Zajonc (1993), participants were asked to assess among different versions of paradigms whether a sign depicted as a representation of a certain word in a Far Eastern alphabet represented a bad or good object, was female or male related, etc. It appeared that affective faces presented before presentation of the sign shaped assessments in affective but not cognitive dimensions.

Words are a common way of evoking emotions in experiments. It has been frequently proven in lexical tasks that the pure emotional charge of a word can influence reaction to it when compared to non-emotional words (Kanske & Kotz, 2012; Schacht & Sommer, 2009; Vinson, Ponari & Vigliocco, 2014). Other emotional traits of word stimuli that influence performance could be valence (Hinojosa, Méndez-Bértolo & Pozo, 2010; Palazova, Sommer & Schacht, 2013), arousal (Yao et al., 2016) and concreteness—the dimension differentiating abstract words from concrete ones (Dreyer et al., 2015; Kousta et al., 2011; Mazzuca et al., 2018; Moseley et al., 2012; Yao et al., 2016). The dimension of concreteness needs some more attention, as some results suggest that the emotive concepts could be related only to abstract words (Villani et al., 2019). We speculate, however, that the dimension of concreteness could be related to origin of stimuli in a more complex way, as abstract concepts are more likely to activate social or linguistic dimensions (Barca, Mazzuca & Borghi, 2017) similarly to reflective emotions, while automatic emotions are related to biological homeostasis and the well-being of an organism, which is more related on concrete objects. It can be concluded that origin is a wide concept that could be part of many conceptualised dimensions of processing, with concreteness as one of them.

It is important to get to know how emotions interact in cognition. The theoretical predictions of the current experiment are based on the dual-mind model of emotion–cognition interactions (Imbir, 2016c). In this model, both emotions and cognition are seen from a dual-mind perspective, indicating that experiential and rational processes may host both domains. Considering interactions between emotional and cognitive processes, we can describe the system-specific ones, particularly the interaction of automatic originated emotions and heuristic processing, or reflective originated emotions and systematic processing. This kind of interaction takes place within just one system of processing and is the reason we may expect that the system-specific relations will, in general, boost both emotional and cognitive processes, which would create a synergy effect based on priming of a cognitive mode by the earlier emotion. Cross-system relations could also occur in which automatic originated emotions may interact with systematic cognition or reflective emotions might interact with heuristic cognition. Such cross-system relations should disturb emotional and cognitive processing, which would be caused by the competition between automatic and reflective modes: earlier emotion coming from one mode should make it more difficult to activate the opposite one. In light of the social cognition domain, we may therefore expect automatic originated emotions to provoke the use of the warmth dimension, as it is strictly related to heuristic cognitive processing. Perceiving a person or an object as warm, which means considering them as friendly, helpful or trustworthy, promotes liking them and the willingness to approach them, which are traits of perception commonly based on heuristics (Fiske, Cuddy & Glick, 2007). Reflective originated emotions, on the other hand, may easily interact with the competence dimension, as it could be understood as the ability to do something or efficacy, traits which are mostly assessed using systematic thinking.

All kinds of emotion–cognition relations, or, from the constructionist view, the emotionally driven processes, should be especially visible in the interpretation and understanding of ambiguous stimuli in which emotions can play a deciding role (Imbir, 2017c). Open tasks, which do not have one specific correct answer and/or have a whole spectrum of possible correct answers, require ambiguity processing (Pulford & Colman, 2007), which is vulnerable to manipulation of the mindset or emotional state (Kempe, Rookes & Swarbrigg, 2013; Raz & Campbell, 2011). Previous research has shown that the emotional properties of words related to valence and origin (automatic vs reflective) affect the processing of an open, ambiguous task (Antosz & Imbir, 2017). In the reflective condition, the latencies of processing the task were longer than in the automatic condition, which leads to the conclusion that the origin of the emotion promoted a certain way of processing the task that was congruous with the origin.

This suggestion was tested in a study on the use of compound signs from the Japanese alphabet introduced as words representing personality traits (Imbir, 2017c). Each presentation of an ambiguous sign was preceded by the presentation of an emotion-laden word, chosen in a way that enabled the contrasting of three levels of valence (negative, neutral and positive) and three levels of origin (automatic, mixed and reflective) in an orthogonal fashion. The task for participants was to remember the word and try to assess whether the sign represented a more competence-related or more warmth-related trait, using a single scale ranging from 1 (competent) to 5 (warm). The results showed that for positively valenced words, automatic originated ones resulted in an assessment of the ambiguous signs as more warm, while reflective originated words resulted in an assessment of the symbols as presenting ideas related to competence. The negatively valenced words appeared not to influence the ambiguous stimuli assessments. The Imbir (2017c) study thus showed the validity of initial expectations. However, due to the use of warmth and competence in a single dimension, the results did not allow for full validation of an independent effect for two distinct dimensions in social cognition (Abele & Wojciszke, 2014; Fiske, Cuddy & Glick, 2007; Imbir, 2017c).

### Aim and hypothesis

The current article presents behavioural data concerning the role of valence (3 levels) and origin (3 levels) in rating ambiguous stimuli on the scales of warmth and competence. First, we expected that the valence of emotion would affect the ratings of warmth but not competence. A negative valence should promote low warmth ratings, while a positive valence should promote high warmth ratings. Second, in accordance with the emotion duality model (Imbir, 2016c, 2017c), we expected to find dissociative effects of origin: that is that automatic origin should promote higher ratings of ambiguous stimuli in terms of warmth rather than competence, while reflective origin should promote higher ratings of ambiguous stimuli in terms of competence rather than warmth. We did not expect the assessments to significantly differ just by the factor of warmth and competence, as both of these dimensions in fact measure positively perceived social traits and there is no reason for the quality of such traits to differ between the ambiguous stimuli.

## Method

### Participants

The study involved 60 participants: 30 women and 30 men. They were students from various Warsaw universities, including the University of Warsaw, the Warsaw School of Economics, the Warsaw University of Technology, Warsaw Medical University and the Warsaw University of Life Sciences, with fields of study that included the humanities, mathematics, natural sciences and technical sciences. The age of the participants ranged from 19 to 26, with an average age of 22 (SD = 1.76).

Based on a previous study (Imbir, 2017c), we concluded that we can expect an effect size ranging from η^2^ = 0.15 to η^2^ = 0.55. A-priori power analyses using the G-Power software (Faul et al., 2007)⁠ based on the effect sizes from the mentioned study showed that a group of 32 participants would be enough to achieve a very high power of 0.95 for the interaction between a within-subject factor (valence or origin) and between-subject factor (type of question), with the same high power for the interaction of both emotional factors, one participant less for the simple effect of one emotion.Based on these analyses, we concluded that the sample size was accurate for the study.

Participation in the study was voluntary. Verbal informed consent was collected from all participants. Data was analysed anonymously and thus we did not collect any personal data (including written consent) that would allow the participants to be identified. All of the participants were native speakers of Polish and had normal or corrected to normal (by glasses or contact lenses) vision. The design, experimental conditions, and procedure were approved by the bioethical committee of the Faculty of Psychology at the University of Warsaw. All of the procedures involving human participants were conducted in accordance with the ethical standards of the institutional and/or national research committee and with the 1964 Helsinki Declaration and its later amendments or comparable ethical standards.

### Design

The study was carried out in a mixed scheme: 3 (valence of the emotional charge of the word: negative vs neutral vs positive; within-subjects factor) × 3 (origin of the emotional charge of the word: automatic vs no particular origin vs reflective; within-subjects factor) × 2 (question concerning dimensions of warmth vs competence, between-subjects factor).

### Procedure

People who expressed their willingness to participate in the study were informed that the study was focused on memory and intuition. They were informed that the survey would be anonymous and that the data received would only be subject to collective analysis. After giving their informed consent to participate in the study, the subjects read the instructions. These explained that their task would consist of memorising the presented words and assessing the words denoting human characteristics coded in the form of QR codes on the dimensions of warmth or competence. Before the actual procedure began, the subjects were also presented with short definitions of ‘warmth’ or ‘competence’. The definition of the warmth dimension was: ‘Warmth means interpersonal relations. Features related to warmth mean that people interact better in a team, have better relationships, are liked and attract other people to each other’. The definition of the competence dimension was as follows: ‘Competence means skills and abilities. Competency traits make people solve problems better, achieve better results, be smarter and be able to rule others’. Next, participants in the study performed an exemplary test task. A single research sample began with exposure to a word lasting 500 ms. The next element was the fixation point, followed by the evaluation of a neutral stimulus (QR code) on the dimension of warmth or competence. The respondents rated on a five-grade scale 270 randomly chosen QR codes from a pool of 400 available in two assessment variants: half of the respondents rated them on the warmth dimension, half on the competence dimension. We used the between-subject method of manipulating the questions about warmth and competence, as we wanted to verify the findings from the previous study that included a plain within-subject model and a bipolar scale of warmth and competence (Imbir, 2017c).

**Figure 1:**
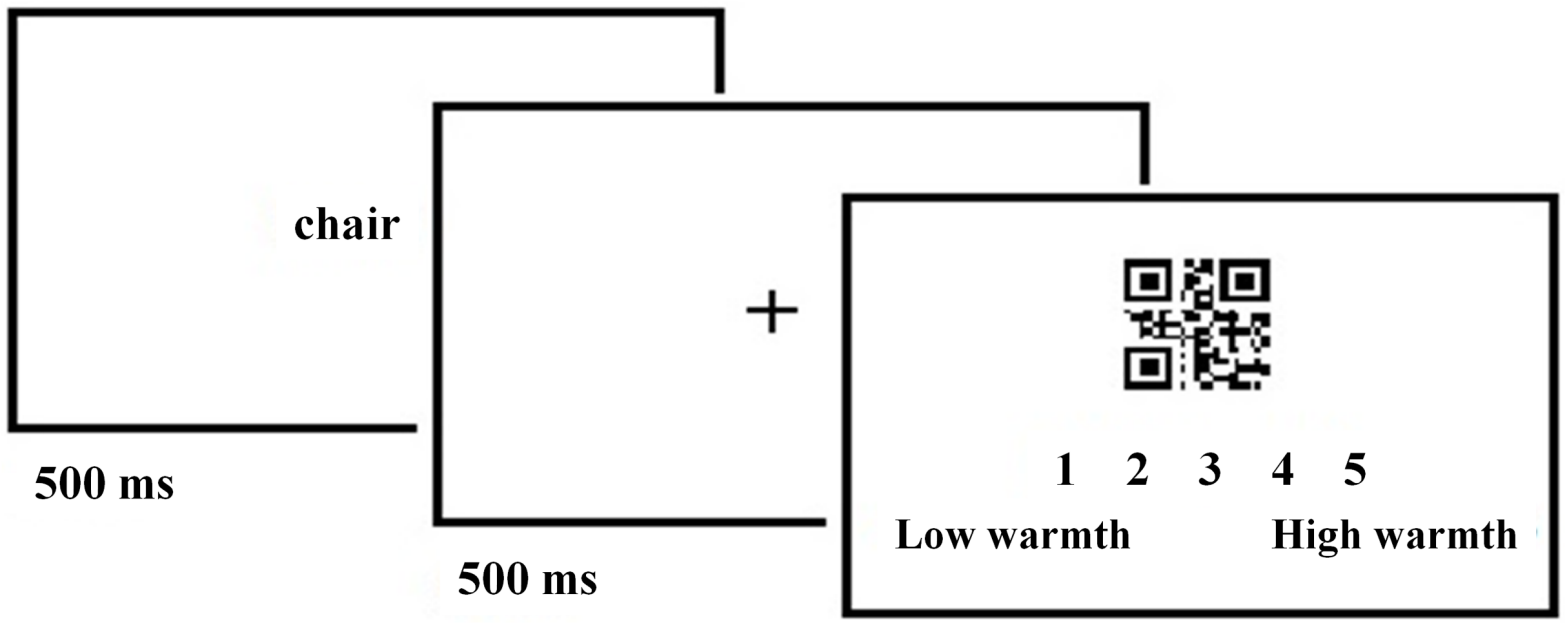
A single trial of the experimental procedure (with warmth as target dimension assessed). The emotional word was presented for 500 ms, then the fixation cross was presented for 500 ms, after which the assessment was done on the warmth or competence scale, time for making the assessment was unlimited. The assessment was done on a scale from 1 (this QR code does not represent the idea) to 5 (this QR code strongly represents the idea).

In the current study, the subjects stated their assessment by pressing keys from 1 (lowest rating) to 5 (highest rating) on a standard laptop keyboard. The ratings the subjects stated and their reaction times were measured. The reaction times were measured from the moment of the onset of the QR code to the moment of pressing the rating key on the keyboard. Times were measured in milliseconds. After completing the examination, participants performed a ‘memory test’ in paper form, consisting of selecting from a set of given words those that, according to the respondent, appeared during the procedure. Finally, the investigator explained the actual goal of the study and answered any questions the participants had. The respondents also had the option of giving the researcher an e-mail address if they were interested in the results of the study. Figure 1 shows a single trial of the experimental procedure.

### Materials

#### Linguistic materials

The stimuli used in our experiment were nouns taken from the Affective Norms for Polish Words Reload database (Imbir, 2016a). This database contains 4,900 Polish words to which normative values based on participant assessments had been assigned. The assessments for each word were made by at least 50 participants, men and women in equal proportion. We chose only nouns with boundary values on the scales of valence and origin to create groups of experimental stimuli, divided as follows: three levels of valence (negative, neutral, positive) and three levels of origin (automatic, no particular origin or reflective), resulting in nine groups in total. There were 15 words in each group. We assumed that variation in the stimuli on certain dimensions should be controlled, as it might influence the processing of words. The controlled dimensions were concreteness, arousal, the frequency of usage in the Polish language (Kazojć, 2011) and the length of the word (number of letters).

Two-way ANOVA analyses were conducted in order to verify the division of the stimuli. The groups divided by the factor of valence differed significantly on the dimension of valence, *F*(2, 126) = 607.44; *p* < 0.001; η^2^ = 0.91; however they did not differ on the dimension of origin, *F*(2, 126) = 1.27; *p* = 0.28; η^2^ = 0.02 or on any controlled dimension. The groups divided by the factor of origin differed on the dimension of origin, *F*(2, 126) = 254.55; *p* < 0.001; η^2^ = 0.80, but there were no differences on the dimension of valence, *F*(2, 126) = 1.88; *p* = 0.16; η^2^ = 0.03 or any controlled dimension. No significant interactions of the factors of valence and origin were found. All the ANOVA results can be found in Appendix 1, as well as the words used in this experiment with exact values on all scales, both manipulated and controlled ones.

#### Ambiguous task

The ambiguous task employed for this experiment was based on assessing a symbol without a specific meaning on one of two scales, namely warmth or competence, the previously described universal dimensions of social cognition. Assessing culturally non-specific symbols is a procedure commonly used for evoking ambiguity (Błaszczak & Imbir, 2012). The stimuli used in this experiment were QR codes. Such symbols are used to code certain links for web-related applications; therefore, it is impossible to understand the meaning of the code without special decoding apparatus (such as a smartphone with a special app). Participants in the experiment understood that each of the codes had a certain meaning, and even though they were unable to decode it, they had to assess the symbol on the scale of warmth or competence, which could be considered as processing in conditions of ambiguity. In each trial, the participants evaluated the QR code on the warmth or competence scale, according to the experimental condition. The exact question was: ‘On a scale from 1 to 5, how much does this symbol represent the idea of warmth/competence?’, where 1 meant the symbol does not represent the idea at all and 5 meant it strongly represents the idea. We assumed that these two dimensions would not be related in any way to the symbols, as they did not have any actual meaning and were randomly generated.

## Results

The results were analysed with respect to two different dependent variables: types of question and reaction times to certain trials. The analysis of variance in the mixed scheme was used: 3 (valence of the emotional word) × 3 (origin of the emotional word) × 2 (question concerning dimensions of warmth vs competence). The within-subject factors were valence and origin of the emotional words, and the between-subject factor was the question (warmth vs competence assessments). Before the analysis, the outlying observations were removed from the data set. We excluded experimental trials that were shorter than 200 ms, which we assumed to be the lowest possible value for a conscious assessment. We also excluded trials longer than three standard deviations from the mean assessment time for subjects. The final sample consisted of 7,524 experimental trials, 92.3% of all recorded trials. For the analysis of the reaction times, we used the same filters as in the preceding analysis. We also converted the raw times measured in milliseconds into natural logarithms due to the right-skewed distribution of raw reaction times (Heathcote, Popiel & Mewhort, 1991).

### Types of questions

We shall start the presentation of the results with the interaction of valence and type of question and origin and type of question because those interactions are the most important ones in the context of our predictions. All the main effects and other interactions are then listed. According to Mauchly’s test, the data did not fulfil the sphericity assumption in the case of valence (χ^2^(2) = 50.03; *p* < 0.001) and origin (χ^2^(2) = 18.34; *p* < 0.001) factors, so the results regarding simple effects for these factors are reported using Greenhouse-Geisser correction (valence: ε = 0.631; origin: ε = 0.784).

**Figure 2:**
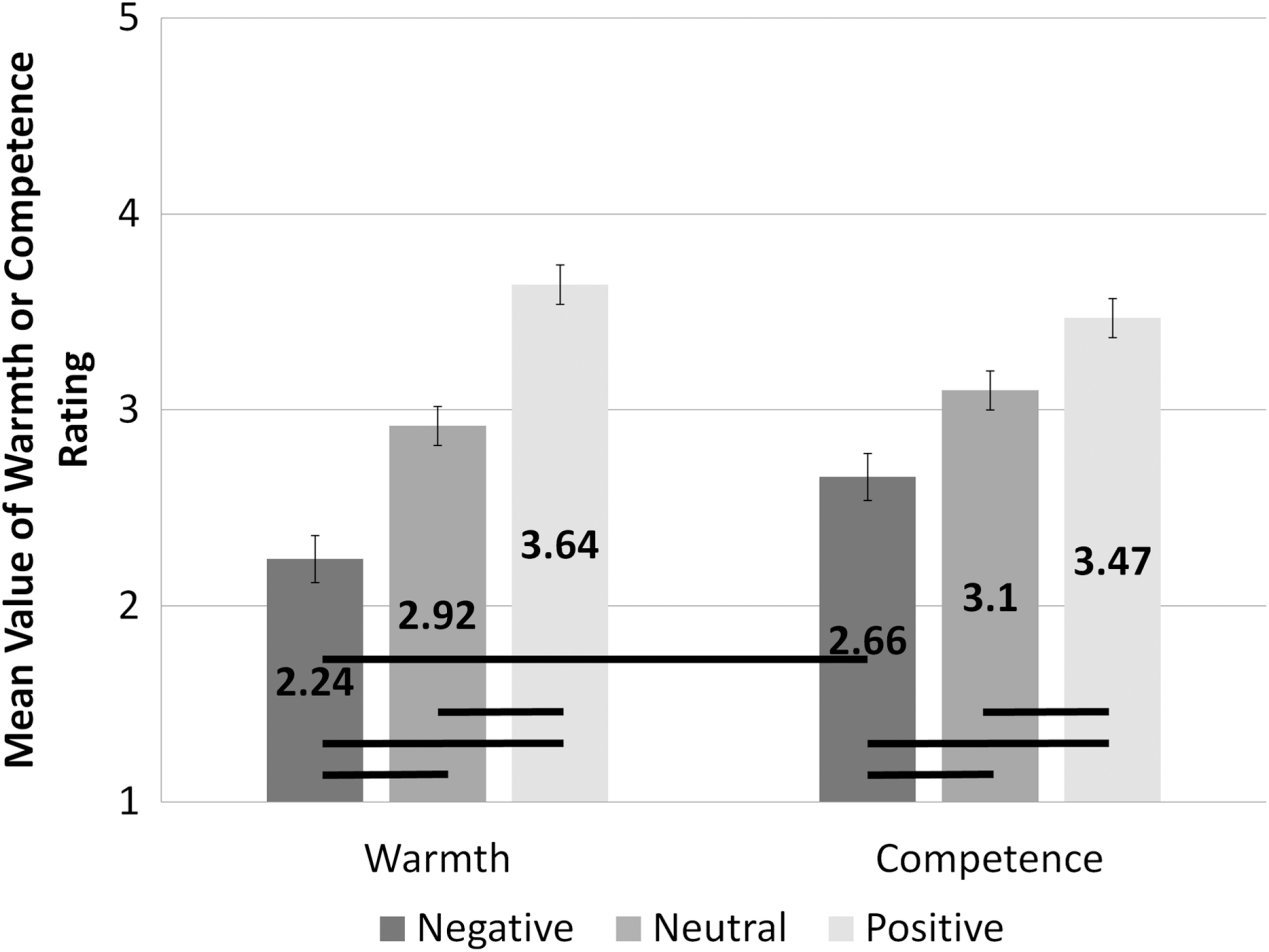
Assessment of warmth and competence for different levels of valence. The bars present mean assessment on a scale from 1 (this QR code does not represent the idea) to 5 (this QR code strongly represents the idea). The assessments made on the warmth scale are presented on the left side of the figure, the assessments on the competence scale are on the right. The bars representing assessments for different levels of emotional valence are filled with different shades of grey. Statistically significant differences are marked with black horizontal lines.

A significant interaction of valence and type of question was observed *F*(1.26, 73.22) = 5.75; *p* = 0.013; η^2^ = 0.09. Analysis of valence differences within the different types of questions with the use of pairwise comparison (with Bonferroni correction) showed that the assessments differed significantly among all conditions of valence. Among the answers to the questions about warmth, we found significant differences between conditions of negative (*M* = 2.24; SEM = 0.12) and neutral valence (*M* = 2.92; SEM = 0.1; *t*(29) = −5.94; *p* < 0.001; *d* = 2.21), negative and positive valence (*M* = 3.64; SEM = 0.1; *t*(29) = −8.28; *p* < 0.001; *d* = 3.08) and neutral and positive valence (*t*(29) = −7.99; *p* < 0.001; *d* = 2.97). In the trials with answers to the questions about competence, we found significant differences between trials with words of negative (*M* = 2.66; SEM = 0.12) and neutral valence (*M* = 3.1; SEM = 0.09; *t*(29) = −4.30; *p* < 0.001; *d* = 1.60), negative and positive valence (*M* = 3.47; SEM = 0.09; *t*(29) = −5.03; *p* < 0.001; *d* = 1.87) and neutral and positive valence (*t*(29) = −4.32; *p* < 0.001; *d* = 1.60). Analysis of assessments for different questions among conditions differing in valence revealed significant differences in the ratings of competence (*M* = 2.66; SEM = 0.12) and warmth (*M* = 2.24; SEM = 0.12; *t*(58) = 2.56; *p* = 0.013; *d* = 0.67) for words of negative valence, but no such difference was found for neutral or positive valence conditions. This interaction is presented in Fig. 2.

**Figure 3:**
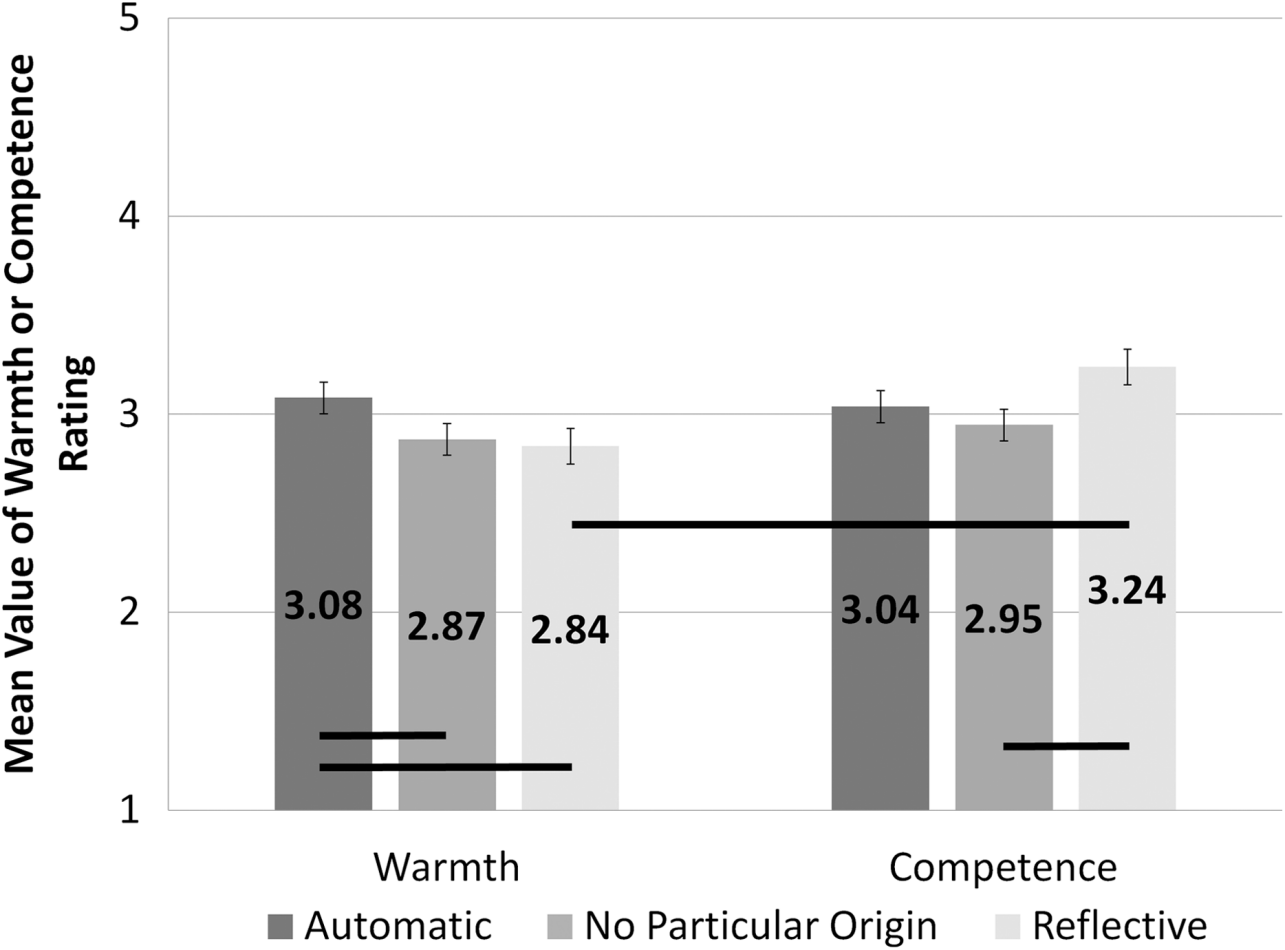
Assessment of warmth and competence for different levels of origin. The bars present mean assessment on a scale from 1 (this QR code does not represent the idea) to 5 (this QR code strongly represents the idea). The assessments made on the warmth scale are presented on the left side of the figure, the assessments on the competence scale are on the right. The bars representing assessments for different levels of origin of emotions are filled with different shades of grey. Statistically significant differences are marked with black horizontal lines.

We also observed a significant interaction between origin and type of question, *F*(1.57, 90.98) = 9.84; *p* < 0.001; η^2^ = 0.15. Analysis of origin differences within the warmth dimension with the use of pairwise comparison (with Bonferroni correction) showed statistically significant differences between trials with words of automatic origin (*M* = 3.08; SEM = 0.08) and those of no particular origin (*M* = 2.87; SEM = 0.08; *t*(29) = 3.99; *p* = 0.001; *d* = 1.48), and between trials with words of automatic origin and reflective origin (*M* = 2.84; SEM = 0.09; *t*(29) = 3.14; *p* = 0.02; *d* = 1.17). There were no significant differences in ratings of warmth between the reflective and no particular origin conditions. In the ratings on competence dimension, a significant difference between conditions of no particular origin (*M* = 2.95; SEM = 0.08) and reflective origin (*M* = 3.24; SEM = 0.08; *t*(29) = −3.37; *p* = 0.001; *d* = 1.25) was found. There were no other significant differences between words of different origin among assessments regarding competence. Among the ratings given in the reflective origin conditions, we revealed significant differences between ratings of competence (*M* = 3.24; SEM = 0.09) and warmth (*M* = 2.84; SEM = 0.09; *t*(58) = 3.21; *p* = 0.002; *d* = 0.84). There were no significant differences between types of question in automatic and no particular origin conditions. These results are shown in Fig. 3.

We observed the main effect of valence, *F*(1.26, 73.22) = 78.38; *p* < 0.001; η^2^ = 0.58. Pairwise comparison of simple effects with Bonferroni correction showed statistically significant differences between ratings given in the negative (*M* = 2.45; SEM = 0.08) and neutral conditions (*M* = 3.01; SEM = 0.06; *t*(59) = −7.21; *p* = 0.001; *d* = 1.88), the negative and positive conditions (*M* = 3.55; SEM = 0.07; *t*(59) = −9.05; *p* < 0.001; *d* = 2.36) and the neutral and positive conditions (*t*(59) = −8.30 *p* < 0.001; *d* = 2.16). The participants rated the QR codes higher on the target dimensions after positive stimuli and lower after negative stimuli, regardless of the exact question.

The main effect of origin was also observed, *F*(1.57, 90.98) = 4.96; *p* < 0.001; η^2^ = 0.08. The assessments given after the words from the group of automatic origin (*M* = 3.06; SEM = 0.06) differed significantly from the assessments given after the words from the group with no particular origin (*M* = 2.91; SEM = 0.06; *t*(59) = 4.03; *p* < 0.001; *d* = 1.05). Ratings stated after words from the group of reflective origin did not differ significantly from ratings stated after words from other groups. No significant effects were found regarding the type of question.

A significant interaction of valence and origin was observed *F*(4, 232) = 3.51; *p* = 0.01; η^2^ = 0.06. Further analysis within the dimension of valence showed significant differences in the condition of negative valence between trials with words of automatic origin (*M* = 2.51; SEM = 0.09) and no particular origin (*M* = 2.33; SEM = 0.09; *t*(59) = 3.46; *p* = 0.003; *d* = 0.90). In the condition of neutral valence, there was a significant difference between trials with words of reflective origin (*M* = 3.12; SEM = 0.08) and no particular origin (*M* = 2.89; SEM = 0.08; *t*(59) = −3.07; *p* = 0.007; *d* = 0.80). In the condition of positive valence, there was a significant difference between trials with words of automatic origin (*M* = 3.65; SEM = 0.08) and no particular origin (*M* = 3.51; SEM = 0.07; *t*(59) = 2.30; *p* = 0.046; *d* = 0.60). Analysis within the dimension of origin revealed significant differences between all groups of words with different valence in all origin conditions. In the automatic origin group, we found a significant difference between trials with negative (*M* = 2.51; SEM = 0.09) and neutral valence (*M* = 2.33; SEM = 0.09; *t*(59) = −6.07; *p* < 0.001; *d* = 1.58), negative and positive valence (*M* = 2.51; SEM = 0.09; *t*(59) = −8.34; *p* < 0.001; *d* = 2.17), and neutral and positive valence (*t*(59) = −7.16; *p* < 0.001; *d* = 1.86). In the group of words with no particular origin, we also found a significant difference between trials with negative (*M* = 3.02; SEM = 0.07) and neutral valence (*M* = 2.89; SEM = 0.08; *t*(59) = −5.83; *p* < 0.001; *d* = 1.52), negative and positive valence (*M* = 3.12; SEM = 0.08; *t*(59) = −9.41; *p* < 0.001; *d* = 2.45) and neutral and positive valence (*t*(59) = −8.07; *p* < 0.001; *d* = 2.10). Subsequently, in the group of words with reflective origin, we found a significant difference between trials with negative (*M* = 3.65; SEM = 0.08) and neutral valence (*M* = 3.51; SEM = 0.07; *t*(59) = −6.31; *p* < 0.001; *d* = 1.64), negative and positive valence (*M* = 3.49; SEM = 0.08; *t*(59) = −7.57; *p* < 0.001; *d* = 1.97) and neutral and positive valence (*t*(59) = −5.24; *p* < 0.001; *d* = 1.36). Positive valence and automatic origin promoted the ambiguous stimuli higher on the universal dimensions of social cognition.

Post-hoc power analyses of observed effects showed that we achieved the power of 0.97 for the interaction of valence and type of question, 0.99 for the interaction of origin and type of question, 0.99 for the simple valence effect, 0.95 for the simple origin effect and 0.86 for the interaction of valence and origin. No significant effect of interaction of all three factors (valence, origin and type of question) was observed.

### Reaction times

**Figure 4:**
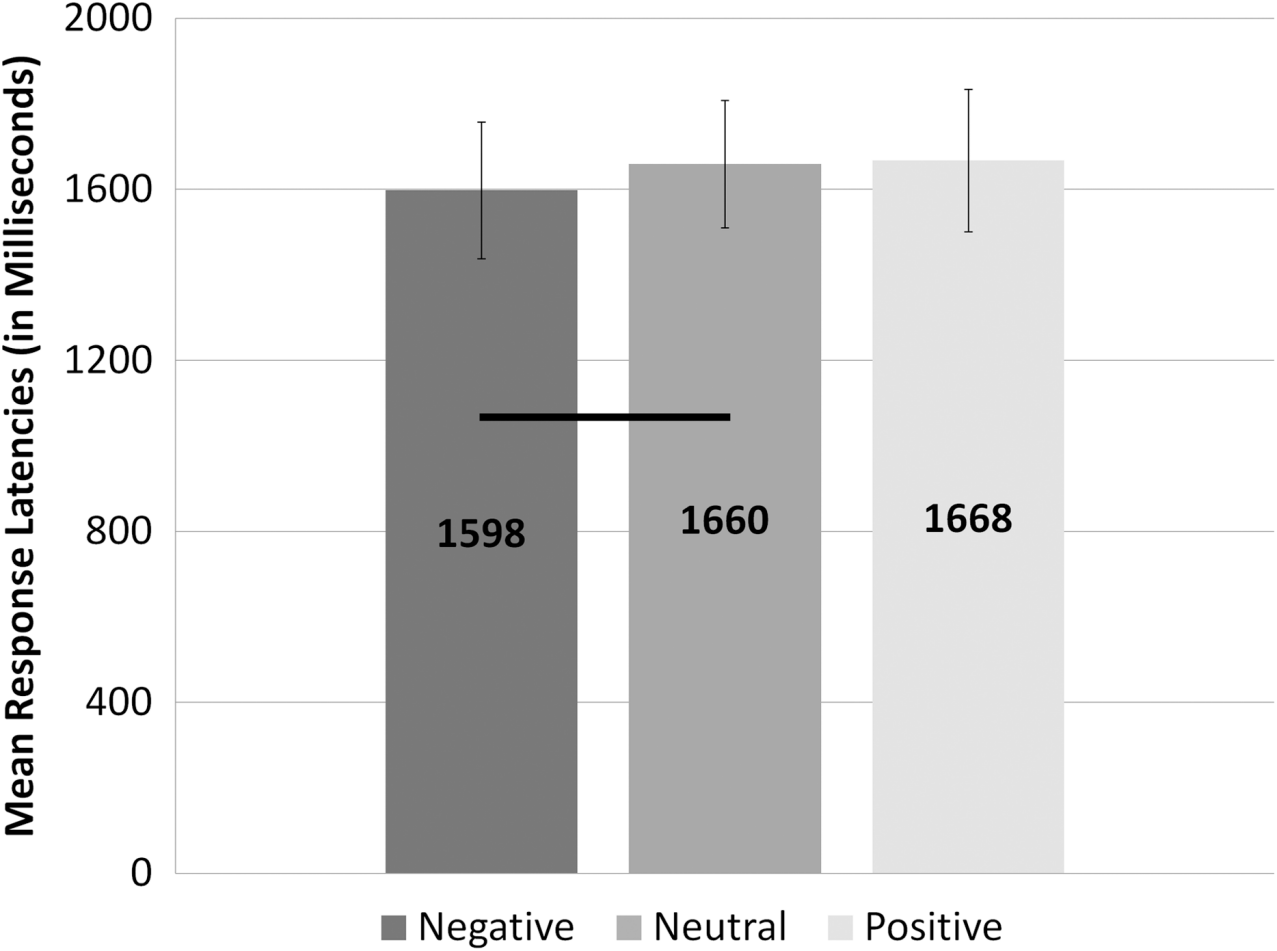
Response latencies to question about warmth or competence for different levels of valence. The bars representing response latencies for different levels of emotional valence are filled with different shades of grey. Statistically significant differences are marked with black horizontal lines.

We revealed the main effect of valence, *F*(2, 58) = 3.56; *p* = 0.015; η^2^ = 0.06 (data for this analysis was consistent with the assumption of sphericity, χ^2^(2) = 3.82; *p* = 0.15; ε = 0.94). Further analysis revealed that the reaction times in trials with words of negative valence (*M* = 1,598.34 ms; SEM = 150.82 ms; LN: *M* = 6.94; SEM = 0.08) were significantly shorter than reaction times in trials with words of neutral valence (*M* = 1,659.5 ms; SEM = 153.71 ms; LN: *M* = 7.00; SEM = 0.08; *t*(59) = −2.90; *p* = 0.014; *d* = 0.76). There were no other significant differences in the condition of valence. These results are presented in Fig. 4.

Post-hoc power analyses revealed that we achieved the power of 0.99 for the effect of valence. Among reaction times, no significant simple effects were observed for factors of origin and type of question. There were no second-level interactions observed between factors nor interactions of all three factors.

## Discussion

### General discussion

The current study aimed to verify the role of valence and origin of an affective state evoked by words in the assessments of ambiguous stimuli in terms of warmth and competence dimensions of social cognition (Abele & Wojciszke, 2014). This issue is important because emotions guide the way we judge objects in our surroundings (Brannon, Sacchi & Gawronski, 2017; Suitner & Maass, 2008) and, therefore, may influence social cognition via the ability to use warmth or competence and not only via direct influence of emotion or mood on cognition that can be triggered by words (Epstein, 2003; Forgas, 1995; Imbir, 2016c; Pessoa, 2008). The detection of such an influence may also be important from the duality-of-mind point of view (Gawronski & Creighton, 2013; Strack & Deutsch, 2014). The connexions between automatic origin of some emotions and warmth, on one hand, and reflective origin of other emotions and competence, on the other hand (Imbir, 2016c, 2017a, 2017c), may imply that both emotions and social cognition are in fact parts of broader mental systems of the experiential and rational mind, as proposed by Epstein (2003) and Imbir (2016c).

Considering the valence of emotional words, we expected to find the effect primarily for participant ratings on the warmth dimension of social cognition. The effect of valence was indeed present in the warmth assessments, but the same effect was also found for competence dimension assessments (cf. Fig. 2). The direction of the effect was in line with our predictions: that is, positive words resulted in more intense assessments of warmth (and competence) than neutral and negative words. This was also congruent with the main effect of valence that was obtained. However, among negative conditions, warmth assessments were lower than competence assessments, that is, the impact of the valence factor on warmth was more pronounced. This partially supports our hypothesis. In the positive condition, assessments of warmth were not found to be more intense than those of competence. In a previous experiment, using a competence–warmth bipolar scale (Imbir, 2017c), no main effect of valence was found but rather the interaction between valence and origin showed origin effect only for positively valenced stimuli. In the current experiment, both negative and positive words influenced the assessments on both scales. Nevertheless, the positive condition influenced assessment of warmth and competence equally strongly, while the negative condition influenced warmth more than competence. This is, in fact, in line with previous findings that show a higher potential for inclusion of contextual valence information in positive conditions than in negative ones (Imbir, 2017c).

As regards origin dimension effects, we predicted that automatic originated words would result in higher warmth-related assessments, while reflective originated words would result in higher competence-related assessments of ambiguous stimuli. This expectation was confirmed. More intense assessments of warmth were observed for automatic originated conditions than for all other conditions, while more intense assessments of competence were observed for reflective conditions than for no specified origin conditions but not for automatic origin conditions. Considering the type of question comparisons, the warmth ratings differ from competence ratings only for reflective originated stimuli. After reading reflective originated words, participants assessed ambiguous stimuli as more competence related than warmth related, but this was not the case for automatic originated stimuli. The whole pattern of results supports our predictions for reflective stimuli and partially for automatic originated stimuli. The lack of expected difference between warmth and competence assessments in automatic originated conditions may be due to the competence assessments that were relatively high. The pattern is consistent with a previous study using the bipolar warmth–competence scale (Imbir, 2017c).

Regarding the reaction times, we found that only the effect of negative words shortened the time of assessments in comparison to neutral and positive words. This is particularly interesting, as shorter times in this condition are tied to lower assessments on both dimensions of social cognition. The effect can be explained as prioritising the processing of negative stimuli. This is especially important in the context of social cognition—if a particular object is perceived in negative categories, it not only influences the warmth and/or competence assessments, it is also very important to assess it quickly, as the object could possibly be threatening. This effect partially replicates the results from our previous study using ambiguous stimuli in which we found negative words promoted faster responses than neutral or positive words (Imbir et al., 2018).

The results of the current experiment are partially in line with the results of a study which showed that warmth is related to approach-avoidance movements, and thus valence, while competence is related to vertical movements (Freddi et al., 2014). In fact, the valence effect in the current experiment appeared stronger for warmth than competence. Some studies (Suitner & Maass, 2008) have suggested that valence is crucial for describing the relations between agency (competence) and communion (warmth). It seems that both are positive, and thus they should correlate positively with one another. This is the case, but when controlling the valence, the correlation turns negative. This suggests that agency and communion are independent dimensions that often contradict each other. Our study supports this claim. Despite the valence effect showing that the more positive the word, the higher the assessment of warmth and competence, when valence is controlled (all origin groups of stimuli were aligned for valence), the positive trend disappears and dissociation of trends can be seen in Fig. 3 (negative for competence, positive for warmth). Thus, each dimension of social cognition should be treated as separate (Fiske, Cuddy & Glick, 2007; Suitner & Maass, 2008; Wojciszke, 2005a, 2005b).

Among the limitations of the current study, we have to list the fact that words used were associated on the semantic level with the competence–warmth bimodal dimension (Imbir, 2017c, 2017a). As was shown in a previous study (Imbir, 2017c), the negative and neutral words were rated as more competence related than warmth related, while positive words were rated as more related to warmth than competence. In the current experiment, positive words actually promoted more intense warmth assessments than negative words. In addition, automatic originated stimuli were assessed as being associated more with warmth than neutral in origin and reflective originated stimuli (Imbir, 2017c). In the current experiment, automatic originated words influenced more intense warmth assessments than reflective originated words, and the opposite effect was present for competence. The above-listed directions of effects suggest that results may be shaped to some extent by the semantic priming of warmth and competence dimensions (Suitner & Maass, 2008). However, the pattern of results is not perfectly suited to semantic priming because the same valence effect was found for competence while the warmth assessments were more intense only for positive conditions, with no higher intensity of competence found for negative stimuli. In addition, the competence assessments were more intense than warmth assessments only for reflective origin, but no reverse difference was found for the automatic condition. This issue is worth investigating; however, it is especially hard to find unrelated stimuli since the fundamental dimensions of social cognition are correlated with valence and origin on a semantic level (Imbir, 2017c; Suitner & Maass, 2008).

We have to note, that the words used in the experiments were related to different ideas and objects, some of them describing inner states (e.g. infatuation), traits (e.g. tolerance), people (e.g. drunk), objects (e.g. goblet) etc. Some of these concepts might have influenced assessments of warmth and competence in a specific way, a good example being the words related to people promoting more radical assessments on the scales, that are directly related to assessing people. This is in an issue that should be controlled in the future studies, identifying other factors shaping social cognition. One of the important factors that may be outlined is the concreteness of the concepts presented by words, however it is difficult to differ it from other emotional dimensions, as it is strongly tied to the emotional aspects of the language (Barca, Mazzuca & Borghi, 2017; Villani et al., 2019). On the other hand, some studies concerning anti-rational aspects of stimuli show that warmth is susceptible to arousal (an experiential mind form of activation) and competence to subjective significance (rational mind form of activation responsible for engagement in a systematic processing) without semantic correlations (Imbir, 2017a).

## Conclusions

The current experiment shows the correspondence between both valence and origin of an emotional reaction to verbal stimuli and assessments of ambiguous stimuli in terms of their relation to the warmth and competence dimensions of social cognition. In general, positive valence results in more intense assessments of both warmth and competence than does negative valence. Automatic origin results in higher assessments of warmth and reflective origin in higher assessments of competence. This study shows that both social cognition and emotions may be interpreted in the context of broader mental systems responsible for experiential and rational processing (Barrett, 2017a, 2017b; Epstein, 2003; Kahneman, 2011; Strack & Deutsch, 2014). Further studies in the field of fundamental dimensions of social cognition and emotions would help us understand how the mind works in terms of dual processes.

## Supplemental Information

10.7717/peerj.10488/supp-1 Supplemental Information 1 Tables including all words used as experimental stimuli, their assessments from the ANPW_R database and statistical analyses of differences between groups of words. Words (in Polish and English) and values are paired with the code of the group as letter and number for manipulated factors: Origin automatic (A, 1) or reflective (R, 3); Valence negative (Neg, 1), neutral (Neu, 2) or positive (Pos, 3) (Sheet 1). Sheet 2 contains mean values of manipulated and controlled factors for each group. Sheet 3 contains results of comparisons between groups (ANOVAs).

## Additional Information and Declarations

### Data Availability

The following information was supplied regarding data availability: Data is available at Figshare: Imbir, Kamil (2020): Can Valence and Origin of Emotional Words Trigger the Interpretation of Ambiguous Stimuli in Terms of Warmth or Competence? figshare. Dataset. DOI 10.6084/m9.figshare.12287789.v1.
